# Fault-Tolerant Scheduling Mechanism for Dynamic Edge Computing Scenarios Based on Graph Reinforcement Learning

**DOI:** 10.3390/s24216984

**Published:** 2024-10-30

**Authors:** Yuze Zhang, Geming Xia, Chaodong Yu, Hongcheng Li, Hongfeng Li

**Affiliations:** College of Computer Science and Technology, National University of Defense Technology, Changsha 410073, China; zhangyuze2025@163.com (Y.Z.); yuchaodong16@nudt.edu.cn (C.Y.); lihongcheng22@nudt.edu.cn (H.L.); lihongfeng22@nudt.edu.cn (H.L.)

**Keywords:** edge computing, fault-tolerant scheduling, graph reinforcement learning, dynamic scenarios, proximal policy optimization

## Abstract

With the proliferation of Internet of Things (IoT) devices and edge nodes, edge computing has taken on much of the real-time data processing and low-latency response tasks which were previously managed by cloud computing. However, edge computing often encounters challenges such as network instability and dynamic resource variations, which can lead to task interruptions or failures. To address these issues, developing a fault-tolerant scheduling mechanism is crucial to ensure that a system continues to operate efficiently even when some nodes experience failures. In this paper, we propose an innovative fault-tolerant scheduling model based on asynchronous graph reinforcement learning. This model incorporates a deep reinforcement learning framework built upon a graph neural network, allowing it to accurately capture the complex communication relationships between computing nodes. The model generates fault-tolerant scheduling actions as output, ensuring robust performance in dynamic environments. Additionally, we introduce an asynchronous model update strategy, which enhances the model’s capability of real-time dynamic scheduling through multi-threaded parallel interactions with the environment and frequent model updates via running threads. The experimental results demonstrate that the proposed method outperformed the baseline algorithms in terms of quality of service (QoS) assurance and fault-tolerant scheduling capabilities.

## 1. Introduction

**Figure 1:**
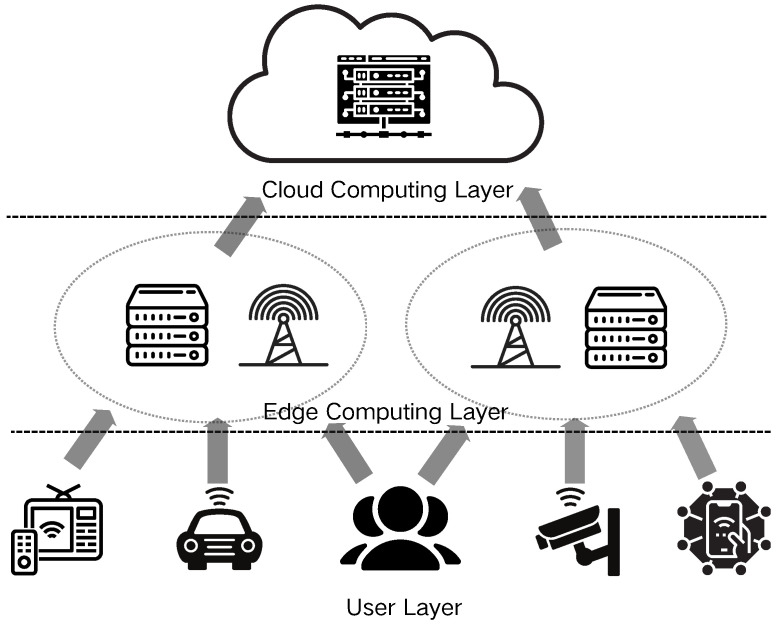
Edge computing architecture.

The rapid proliferation and widespread adoption of Internet of Things (IoT) technologies have exposed the limitations of traditional centralized cloud computing models in meeting escalating demands for data processing and real-time capabilities [1,2]. Edge computing, an innovative distributed computing paradigm, strategically positions computing resources and data storage proximate to the network edge (in close proximity to data sources or users), thereby significantly mitigating data transmission latency and enhancing system responsiveness and reliability. As shown in Figure 1, this architecture facilitates more efficient task management. Consequently, edge computing has emerged as a critical technological foundation across diverse domains, including smart cities, autonomous vehicles, and industrial Internet applications [3,4].

Nevertheless, the constraints imposed by limited communication resources at the user end and cost considerations necessitate judicious utilization of resources within the edge network. It is imperative to optimize user service capabilities through the effective allocation of these finite communication resources. Thus, the establishment of an efficient offloading scheduling framework between end computing nodes and servers is essential. Such a framework facilitates intelligent decision making for the transfer and execution of computing tasks among devices via communication links, thereby optimizing system overhead and maximizing user satisfaction.

The edge computing environment is characterized by high heterogeneity and dynamism, with computing nodes potentially dispersed across diverse geographical locations and exhibiting substantial variations in hardware configurations, computational capacities, and network bandwidths. Furthermore, edge devices frequently operate under resource constraints and are susceptible to external environmental factors, resulting in a higher incidence of device failure. Consequently, achieving efficient and reliable task scheduling in this complex and volatile environment has emerged as a critical challenge in edge computing research. The implementation of a robust, fault-tolerant mechanism is thus crucial to ensure stable execution and efficient scheduling of edge computing tasks.

Conventional fault-tolerant scheduling methodologies often rely on static models, which lack the requisite adaptability to dynamic system changes and struggle to effectively manage the frequent faults and resource fluctuations inherent in edge computing environments [5]. Recent advancements in machine learning and artificial intelligence have underscored the potential of graph reinforcement learning (GRL) [6] as a powerful tool for addressing complex network structural data and dynamic optimization challenges. GRL demonstrates the capability to capture intricate inter-nodal relationships and adapt to evolving system states by leveraging reinforcement learning techniques on graph structures, thereby enabling intelligent and dynamic optimization of task scheduling processes.

This paper proposes a novel fault-tolerant scheduling mechanism for dynamic edge computing scenarios, predicated on graph reinforcement learning. The mechanism employs a graph reinforcement learning algorithm to establish relationships between tasks and computing nodes, dynamically perceive and anticipate system state changes, intelligently adjust task scheduling policies, and facilitate expeditious task rescheduling in the event of equipment failure or resource insufficiency. This approach ensures high system availability and maintains an elevated task completion rate.

The primary contributions of this research are as follows:

(1) Graph Construction: We develop a graph-based representation which captures the complex dependencies between tasks and compute nodes, as well as dynamic changes in system states. The state is modeled using a graph neural network (GNN), with dynamic optimization of the graph achieved through reinforcement learning algorithms.
(2) Graph Reinforcement Learning Model: We formulate a reinforcement learning model based on graph neural networks to effectively manage the complex relationships between tasks and nodes while adapting to dynamic changes in system states.
(3) Fault-Tolerant Scheduling Strategy: We design a fault-tolerant scheduling strategy leveraging the graph reinforcement learning algorithm to monitor the node status and task progress in real time during execution, automatically detect faults, and initiate task rescheduling, thereby enhancing system reliability and resource utilization.
(4) Experimental Validation: We conduct extensive simulations to empirically validate the superiority of the proposed mechanism in terms of fault-handling capability, scheduling efficiency, and task completion rate compared with traditional methodologies.

The remainder of this paper is structured as follows. Section 2 provides a concise overview of the current research landscape in fault-tolerant scheduling within edge computing environments. Section 3 elucidates the system architecture and computational communication model adopted in this study, and we formulate optimization problems based on the system model. Section 4 introduces the fault-tolerant scheduling method predicated on asynchronous graph reinforcement learning. Section 5 presents and analyzes the experimental results. Finally, Section 6 offers concluding remarks and summarizes the key findings of the research.

## 2. Related Work

In recent years, task scheduling and fault tolerance mechanisms in edge computing have garnered significant research attention. This section provides a comprehensive review of existing research, focusing on three key aspects: traditional scheduling mechanisms, heuristic-based scheduling strategies, and machine learning-based fault tolerance mechanisms [7].

### 2.1. Traditional Scheduling Mechanisms

Traditional scheduling mechanisms represent early explorations in edge computing task scheduling, primarily relying on static models and classical algorithms for task allocation and management. These approaches typically consider task resource requirements and node load but often lack effective mechanisms for addressing dynamic changes and fault handling.

DEFT incorporates uncertainty into task runtime estimation models and proposes a fault-tolerant task assignment mechanism which strategically employs two fault-tolerant task scheduling models while considering uncertainty [8]. Additionally, DEFT utilizes an overlap mechanism to enhance resource utilization in cloud environments. Building upon these two mechanisms, the authors propose an innovative, dynamic fault-tolerant elastic scheduling algorithm for real-time task scheduling in cloud environments, where system performance fluctuations are a significant consideration.

DFTLA introduces a novel, dynamic fault-tolerant learning automaton task scheduling method [9]. This approach determines task assignments to fog nodes based on variable structure-learning automata. Experimental results demonstrated that the algorithm optimizes the response time and energy consumption while ensuring reliable task execution.

While traditional scheduling mechanisms perform adequately in deterministic task environments and stable conditions, their limitations become increasingly apparent in highly dynamic and heterogeneous edge computing environments. Consequently, researchers are increasingly focusing on more intelligent and adaptive scheduling strategies.

### 2.2. Heuristic-Based Scheduling Strategies

In contrast to traditional static scheduling strategies, heuristic-based fault-tolerant scheduling approaches offer enhanced flexibility and adaptability, enabling systems to maintain stability and service reliability in dynamically changing environments.

Zhang et al. proposed the enhanced whale optimization algorithm (EWOA) for cloud task scheduling [10]. By integrating Lévy flight with the traditional whale optimization algorithm, the EWOA expands the search space and accelerates convergence. Implemented using CloudSim, the EWOA demonstrates superior performance compared with existing algorithms in terms of resource utilization, energy consumption, and execution cost, effectively addressing multi-objective scheduling challenges.

Wang et al. introduced FASDQ, a fault-tolerant adaptive scheduling mechanism with dynamic quality of service (QoS) awareness for edge containers handling delay-sensitive tasks [11]. FASDQ extends the primary (backup) model by applying QoS on demand to task copies and incorporates a resource-adaptive adjustment mechanism. Simulations conducted on the EdgeCloudSim platform demonstrated that FASDQ reduces the execution time and improves reliability and resource utilization compared with alternative methods.

Luo et al. proposed a simulation driver framework based on real cloud system operation logs to enhance the fault tolerance of large-scale cloud computing systems [12]. The innovation lies in the combination of a Markov model with a reliability-conscious resource scheduling algorithm to economically and effectively improve system reliability.

While heuristic-based fault-tolerant scheduling strategies enhance resource utilization and reduce costs, they often yield suboptimal solutions and incur high computational overhead. These methods struggle to adapt to dynamic cloud environments and frequently require extensive parameter tuning, limiting their effectiveness in real-time applications. Improving their optimization capabilities and adaptability is crucial to better meeting the demands of modern cloud computing environments.

### 2.3. Machine Learning-Based Fault Tolerance Mechanisms

The advancement of machine learning technologies has prompted researchers to explore their application in edge computing task scheduling, aiming to enhance the intelligence and adaptability of scheduling mechanisms. Machine learning-based scheduling strategies leverage historical system operational data to predict future resource demands and node statuses, thereby achieving more efficient task allocation [13].

### 2.4. Heuristic-Based Scheduling Strategies

Tuli et al. developed PreGAN+ [14], a semi-supervised fault prediction and migration model designed for dynamic mobile edge environments. By utilizing generative adversarial networks (GANs), PreGAN+ predicts node contention and resource overloads, generating proactive migration decisions to mitigate downtime. The integration of semi-supervised learning with a Transformer-based network enables PreGAN+ to outperform state-of-the-art methods in terms of fault detection and quality of service (QoS) metrics.

Kalaskar et al. proposed a dual Q-learning approach coupled with a dynamic fault-tolerant real-time scheduling algorithm (DFTRTSA) [15]. Their method employs double deep Q-learning to optimize scheduling based on system conditions and error states, thereby enhancing fault tolerance and resource management. Their experimental results demonstrated that DDQL-DFTRTSA significantly improves defect tolerance, energy efficiency, and real-time performance compared with traditional methods.

Yan et al. presented a fault-tolerant scheduling framework for heterogeneous UAVs in IoT data collection applications [16]. They enhanced data collection reliability using UAVs by introducing a utility-based fault tolerance model (UBFT) which balances reliability and efficiency. By modeling UAV scheduling as a multi-objective optimization problem, they proposed the ACTOR method to optimize data throughput and load balancing. Both simulations and real-world tests validated ACTOR-UBFT’s superior fault tolerance and performance.

Chen et al. have made significant contributions to blockchain-enabled mobile crowdsensing (MCS) systems. In their *IEEE Communications Magazine* paper [17], they introduced a deep reinforcement learning (DRL) framework for intelligent offloading of computation-intensive proof-of-work (PoW) tasks to edge servers, achieving lower latency and power consumption. Their subsequent work in future generation computer systems [18] presented a consortium blockchain framework incorporating a novel credit-based PoW (C-PoW) algorithm to enhance reliability while reducing PoW complexity. Additionally, they proposed a scalable deep reinforcement learning-based computation offloading (DRCO) method, combining proximal policy optimization (PPO) and a differentiable neural computer (DNC). This approach effectively optimizes offloading decisions and demonstrates superior performance across various scenarios compared with state-of-the-art methods.

Despite these advancements, existing machine learning-based fault-tolerant scheduling methods face several challenges. They often struggle to capture complex node interdependencies, leading to suboptimal decisions in dynamic environments. The high computational demands of neural networks and reinforcement learning algorithms limit real-time responsiveness and scalability in large-scale edge systems. Moreover, the need for extensive parameter tuning and limited robustness across diverse scenarios hinder practical deployment. These limitations underscore the necessity for more efficient and adaptable scheduling approaches, such as the proposed asynchronous graph reinforcement learning model, which aims to better capture node relationships and improve real-time performance in dynamic edge environments.

In Table 1, we can see that while machine learning-based methods exhibit better adaptability and scalability, they also entail higher computational overhead and complexity in implementation.

## 3. Systems and Computational Models

This section introduces fault-tolerant scheduling in edge computing scenarios, simulates the instability of real-world computing environments by incorporating task processing uncertainty, and proposes an optimization objective which measures user quality of service (QoS) through task processing latency.

### 3.1. Computing Scenario

**Figure 2:**
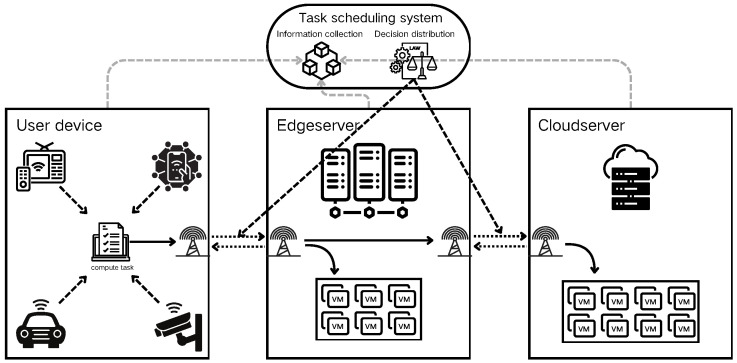
Task scheduling model.

The research presented in this paper addresses common edge computing scenarios. As illustrated in Figure 2, these scenarios primarily comprise user devices, edge servers, cloud servers, and scheduling centers. These components are interconnected via communication links and are geographically distributed. In the user scenario, computing tasks are generated by devices and wirelessly transmitted to servers for processing. The dispatching center aggregates environmental information to manage the transmission process of computing tasks.

In our problem model, computing tasks originate from IoT devices. These tasks are either processed locally or transmitted to servers via communication links, based on decisions made by the dispatching center. The computational results are then returned to the originating device. Given that most IoT scenarios are latency-sensitive, this study focuses primarily on the task processing time. We considered the finest granularity, treating each task as an indivisible unit.

Furthermore, we incorporated the instability factors of computing equipment in the fault tolerance problem, including fluctuations in the estimated computation time and the possibility of calculation failure. Consequently, we modeled each computational task as a triplet [$\alpha_{i}$, $\beta_{i}$, $\gamma_{i}$], where $\alpha_{i}$ represents the data volume required for computation task *i* (typically measured in bits), $\beta_{i}$ denotes the clock cycles required for computation task *i* (usually in Gcycles), and $\gamma_{i}$ indicates the task priority, quantifying the task’s importance.

### 3.2. Edge Computing Model

We posit the existence of *n* available IoT devices in the current edge computing scenario. Given that this study focuses on fault-tolerant scheduling of task runtimes, with the device processing time serving as a metric, each device is modeled as $V_{k}$ with parameter information [$f_{k}$, $\omega_{k}$, $\varphi_{k}$]. Here, $f_{k}$ represents the computing capability of local computing device *k*, $\omega_{k}$ denotes the uncertainty associated with tasks processed by device *k*, and $\varphi_{k}$ indicates the failure rate of tasks processed by device *k*. Additionally, we employ $R_{m,n}$ to denote the transmission rate of the network link from device *m* to device *n* and $\omega_{m,n}$ to represent the uncertainty in the transmission rate from device *m* to device *n*.

Given the relatively small volume of calculation result data and high downlink data rates, the latency in returning calculation results is typically negligible. As tasks are subject to transmission link instability during transfer, the actual transmission time $T_{i}^{m,n}$ required for task *i* to be transmitted from device *m* to *n* can be expressed as follows:

(1) $$T_{i}^{m,n}=\left[\left(1-\omega_{m,n}\right)\alpha_{i}/R_{m,n},\left(1+\omega_{m,n}\right)\alpha_{i}/R_{m,n}\right],$$

Since a task may traverse multiple network links before reaching its target device, $T_{i}^{trans}$ represents the total time required for task *i* in transit. Thus, $T_{i}^{trans}$ can be expressed as follows:

(2) $$T_{i}^{trans}=\sum_{\left(m,n\right)\in E_{i}}T_{i}^{m,n},$$

where $E_{i}$ is the set of network link edges traversed by task *i*.

Given the dynamic real-time scheduling scenario under investigation, it is essential to describe the processing lifecycle of tasks. We define $t_{i}$ as the task generation time, $T_{i}^{dec}$ as the decision system runtime, $T_{i}^{trans}$ as the task transfer time, and $T_{i}^{wait}$ as the task waiting time. Consequently, the actual time at which task *i* commences processing on device *k*, denoted as $t_{k,i}$, is

(3) $$t_{k,i}=t_{i}+T_{i}^{dec}+T_{i}^{wait}+T_{i}^{trans},$$

The time $T_{k,i}$ required for computing task *i* on device *k* can be expressed as follows:

(4) $$T_{k,i}=\beta_{i}/f_{k}.$$

Due to device heterogeneity, the actual computing time may fluctuate, and task processing may fail. Therefore, the actual processing time $T_{k,i}^{actual}$ of the computing task is

(5) $$T_{k,i}^{actual}\in \left[\left(1-\omega_{k}\right)T_{k,i},\left(1+\omega_{k}\right)T_{k,i}\right],$$

where $\omega_{k}$ is the fluctuation coefficient of the calculation time, which is related to device stability. Additionally, we define an error rate $\varphi_{k}$, representing the probability of calculation interruption due to errors during execution.

Assuming $T_{k,i}^{actual}$ represents the sample space of all possible real runtimes, with the actual runtime being a sample point, and assuming the runtime follows a uniform distribution [DEFT], the probability density function of $T_{k,i}^{actual}$ can be expressed as follows:

(6) $$f\left(T_{k,i}^{actual}\right)=\left\{\begin{array}{cc} \frac{1}{2\omega_{k}T_{k,i}}, & \mathrm{if}\;\left(1-\omega_{k}\right)T_{k,i}<T_{k,i}^{actual}<\left(1+\omega_{k}\right)T_{k,i};\\ 0, & \mathrm{otherwise}. \end{array}\right.$$

The completion time $t_{k,i}^{finish}$ of calculation task *i* is

(7) $$t_{k,i}^{finish}=t_{k,i}+T_{k,i}^{actual}.$$

The timeout period $T_{k,i}^{timeout}$ of task *i* is defined as

(8) $$T_{k,i}^{timeout}=\left\{\begin{array}{cc} \max(0,t_{k,i}^{finish}-t_{i}^{deadline}), & \mathrm{with}\;\mathrm{probability}\;1-\varphi_{k};\\ \kappa_{k,i}, & \mathrm{with}\;\mathrm{probability}\;\varphi_{k}. \end{array}\right.$$

Due to the possibility of calculation failure during task execution, preventing results being returned, it is necessary to establish a timeout upper limit $\kappa_{k,i}$.

Consequently, the final calculation result may manifest in the following scenarios:

1. If the task is successfully executed, and $\left\lceil t_{k,i}^{finish}\right\rceil <t_{i}^{deadline}$, then the task is guaranteed to not time out;
2. If the task is successfully executed, and $\left\lfloor t_{k,i}^{finish}\right\rfloor <t_{i}^{deadline}<\left\lceil t_{k,i}^{finish}\right\rceil$, then there is a probability of task timeout;
3. If the task is successfully executed, and $t_{i}^{deadline}<\left\lfloor t_{k,i}^{finish}\right\rfloor$, then the task is certain to time out;
4. If the task fails to execute, then the task timeout is $\kappa_{k,i}$.

### 3.3. Problem Formulation

The central problem addressed in this paper can be defined as the minimization of the weighted sum of user task timeout times, which can be formally expressed by the following equation:

(9) $$\min\sum_{i\in N}\gamma_{i}T_{K,i}^{timeout},$$

where N represents the set of all computing tasks.

Our objective is to minimize the total weighted timeout time by optimizing the offloading path and scheduling policy for each task.

## 4. Enhancement of Fault-Tolerant Scheduling Mechanism Based on Graph Reinforcement Learning

This section presents a detailed exposition of the fault-tolerant scheduling mechanism utilizing proximal policy optimization (PPO)-based graph reinforcement learning [19]. The discussion encompasses the process of modeling edge computing scenarios as graph data, the formalization of task scheduling problems, and the specific update mechanisms and algorithmic concepts underlying fault-tolerant scheduling policies.

### 4.1. Graph Modeling

The inherent complexity of edge computing environments presents a fundamental challenge in the development of fault-tolerant scheduling mechanisms based on graph reinforcement learning. Edge computing is characterized by dispersed resources, heterogeneous nodes, and unstable network connections. These attributes render traditional computing models and scheduling strategies ineffective, as they typically assume a relatively homogeneous and stable computing environment, a stark contrast to the multifaceted reality of edge computing. Consequently, graph modeling becomes an essential approach capable of more accurately representing the intricacies of the edge computing environment, including the distribution and availability of diverse computing resources.

Furthermore, the intricate dependencies between edge computing tasks and resources pose an additional challenge in implementing effective scheduling policies. In edge computing scenarios, specific tasks may require execution on particular nodes to meet stringent latency requirements. Simultaneously, data transfer between certain nodes may be significantly impacted by network bandwidth limitations or latency issues. In this context, graph models offer a more intuitive and precise representation of the complex relationships between various computing nodes, tasks, and resources. This enhanced representation facilitates the optimization of task scheduling and resource allocation strategies, thereby improving the overall efficiency and effectiveness of the edge computing network.

**Figure 3:**
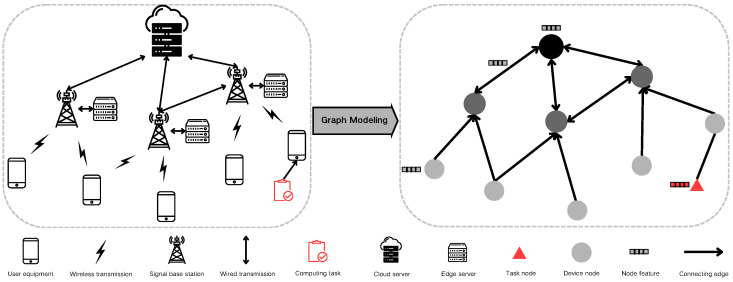
Graph modeling process.

The process of modeling edge computing scenarios as graph data necessitates mapping various system components to graph elements. Initially, we represent device nodes in the system, such as edge servers and Internet of Things (IoT) devices, as graph nodes, symbolizing the computing resources within the scenario. The network connections between computing devices are then mapped as edges connecting the nodes in the graph data. Figure 3 illustrates this specific conversion methodology.

Moreover, the parametric information of devices and networks must be translated into characteristic values within the graph data. For instance, the computing power, fluctuation coefficient, and failure rate of computing devices are calculated and represented as node characteristic values in the graph data. Similarly, network connections between devices, including bandwidth and latency, are represented as edge characteristic values in the graph data.

The characteristic values for computing devices are denoted as [$f_{K}$, $\omega_{k}$, $\varphi_{k}$], representing the computing capacity, calculation fluctuation coefficient, and calculation error rate, respectively. For computing tasks, the characteristic values are [$\alpha_{i}$, $\beta_{i}$, $\gamma_{i}$, $t_{i}^{gen}$, $t_{i}^{deadline}$], representing the data volume, computation amount, task priority, creation time, and deadline, respectively. The transmission link characteristics are represented by [$R_{m,n}$, $\omega_{m,n}$], denoting the transmission rate and transmission volatility, respectively.

### 4.2. Scheduling Problem Formulation

In dynamic edge computing scenarios, the strategy to achieve fault-tolerant scheduling must adapt to highly variable conditions. The spatial structure and parameter information of graph data may change rapidly, yet the core task processing remains fixed. Tasks emerge randomly on IoT devices, with some transmitted to edge or cloud servers through our scheduling strategy. These servers process the tasks and return results to the IoT devices. The generation of scheduling policies adheres to the Markov property, meaning the current policy only considers recent system state information, disregarding longer historical environmental data. Thus, we can describe it as a Markov decision process (MDP) represented by the tuple M={ST,A,P,R}, where the following definitions apply:

- ST denotes the system state;
- *A* represents the action space;
- *P* signifies the transition probability (e.g., $P_{st,st^{\prime }}^{a}=P\left[ST_{t+1}=st^{\prime }|ST_{t}=st,A_{t}=a\right]$ indicates the transition probability from state st to $st^{\prime }$ after action *a*);
- $R_{st}^{a}=E\left[R_{t+1}|ST_{t}=st,A_{t}=a\right]$ represents the immediate reward for taking action *a* in state st;

The objective of the reinforcement learning model is to determine a policy $\pi \left(a|st\right)=P\left[A_{t}=a|ST_{t}=st\right]$ which maximizes the reward, enabling optimal action selection across various states.

State: In this study, the state is defined as comprehensive information about the system at a given time point, encompassing: the following

- The status of each compute node (e.g., CPU);
- The current task queue;
- Network conditions (e.g., bandwidth utilization, and latency);
- Environmental parameters closely related to system performance;

This state definition aims to fully capture the system’s operation at any given moment, providing a sufficient informational basis for action selection. By incorporating multifaceted state information, we ensure that the reinforcement learning model can make optimal decisions based on a holistic view of the current system state.

Action: Given the context of fault-tolerant scheduling in dynamic edge computing scenarios, we define the action space of the model to include task scheduling and fault-tolerance decisions. Specific actions comprise the following:

- Task transfer: Migrating computing tasks from the current node to one or more nearby computing devices;
- Local computation: Executing tasks on the current node without migration;
- Backup computation: Simultaneously sending tasks to multiple nodes for processing to mitigate potential node failures;
- Waiting: Delaying task execution while awaiting improved network conditions or computing resources;
- Task discard: Abandoning certain tasks when system load is excessive or task completion within specified time constraints is infeasible to enhance overall system performance;

Action selection is contingent upon the current system state, with the reinforcement learning model’s strategy aimed at minimizing total task delay and improving overall system performance.

Reward: As the optimization goal is to maximize service quality by minimizing the total weighted task computation delay, we define the reward as the negative value of the task delay. Rewards are only provided upon task completion, with no interim rewards given during the scheduling and transmission process.

### 4.3. Model Architecture

Given that the scenario is modeled using graph data, graph neural networks are employed for processing. We utilize a custom graph neural network layer based on the message passing neural network (MPNN) framework [20], which primarily implements the following:

1. Feature normalization: L2 normalization is applied to both node and edge features to mitigate the adverse effects of device parameter discrepancies on model performance.
2. Self-loops and information aggregation: Self-loops are introduced to enhance the model’s capability to update information, particularly for nodes with low connectivity. Edge and node features are combined to compute an intermediate edge representation, which is used for updating node features.
3. Attention mechanism: An attention mechanism is incorporated to enable the model to focus on important nodes within the graph. The computation of attention scores allows the model to consider information from different neighbors in a weighted manner during node feature updates.
4. Feature update: Different multi-layer perceptrons (MLPs) are used for processing, supporting the feature updating of heterogeneous nodes based on their roles.

Actor: The actor consists of a two-layer graph neural network. The first layer primarily captures local node features, while the second layer further integrates these features to learn a global state representation. This architecture enables the actor to comprehensively understand and interpret complex environmental state information for more accurate action predictions. The actor network’s output is a probability vector over all possible actions, allowing for probabilistic action selection which promotes environmental exploration while favoring actions with high expected returns.

Critic: The critic’s structure parallels that of the actor, employing a two-layer graph neural network. It receives the state as input but outputs a one-dimensional value representing the current state’s value. The critic evaluates the expected cumulative return from a given state under the current policy, expressed as a value function. This evaluation guides actor updates, steering the policy toward improved long-term returns. Like the actor, the critic’s first layer captures local structural features, while the second layer integrates these to learn a global representation.

### 4.4. Update Process

This study adopts the proximal policy optimization (PPO) method for reinforcement learning in dynamic edge computing scenarios, utilizing neural networks to accommodate complex state and action spaces. PPO enhances training stability and efficiency by moderating policy update steps through a clipping mechanism, preventing abrupt policy changes and ensuring consistent learning. Both the policy (actor) and value (critic) functions are modeled with neural networks, with the actor outputting action probabilities for each state and the critic estimating the current state’s value.

PPO, an actor-critic-based reinforcement learning algorithm, combines the value function $V(s,\theta_{critic})$ with the policy function $\pi (a|s;\theta_{actor})$ to optimize long-term rewards. The critic network evaluates action values using an n-step temporal difference error, while policy updates in the actor network incorporate a clipping operation to limit policy adjustments, enhancing update safety and stability.

The update strategy, which is particularly suitable for dynamic edge computing, integrates multi-threaded sample generation and a sliding window for sample averaging, accelerating sample collection and smoothing data variance. This approach ensures PPO’s adaptability and superior performance in rapidly changing environments.

**Figure 4:**
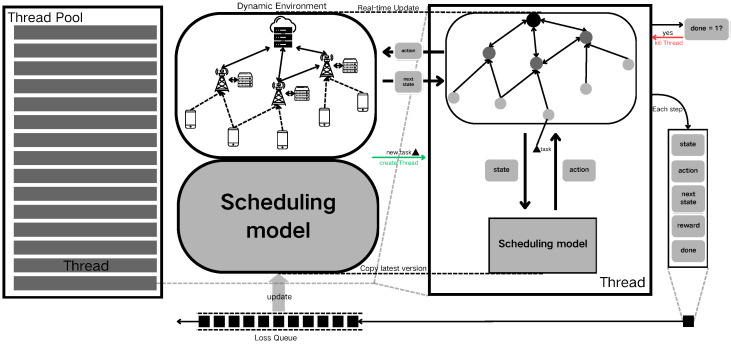
Dynamic update process.

Figure 4 shows the dynamic update process. Before initiating the dynamic scenario, a thread pool is established to manage sub-threads responsible for interacting with the environment. The device information and communication data within the dynamic edge computing environment are modeled as graph data. Concurrently, the fault-tolerant scheduling model is initialized as the primary reinforcement learning model. Once the dynamic environment is up and running, each time a new computational task arrives, a snapshot of the current environment, including the new task, is created. Additionally, a duplicate of the fault-tolerant scheduling model agent is spawned and assigned to a thread for processing.

Within each thread, the current state of the environment is encapsulated and fed into the fault-tolerant scheduling model to determine the appropriate actions. The environment functions utilize these actions to schedule the computational tasks, subsequently updating the environmental parameters, such as the feature values of the graph data. For instance, if an action involves migrating a computational task from user node *i* to edge server node *j*, then the earliest available time for the corresponding communication link is adjusted accordingly. The updated environmental information is then returned as the next state. Simultaneously, the system calculates the reward associated with the action based on the dynamic scenario, using the negative value of task timeouts as the reward, which is then input into the scheduling model for optimization. The state, action, next state, reward, and completion signal are bundled together as update information for the primary model and placed into a model loss update queue. When multi-step execution reaches a terminal state, the thread is immediately terminated, and system resources are reclaimed.

As the dynamic scenario progresses, the update information generated by the threads accumulates in the model loss update queue. When the conditions for updating the primary model are met, a specified number of the most recent samples from the sample storage queue are used to perform an update on the primary model. In subsequent dynamic task processing, new sub-threads are created by copying the parameters of the primary model, ensuring that each thread operates based on the latest scheduling strategies.

## 5. Performance Evaluation

In this section, the performance of the proposed algorithm will be tested by simulating dynamic edge computing scenarios, including the instability of transmission and calculation in the simulation scenario, as well as the variation in communication relations, and the fault tolerance performance of the algorithm will be tested. In addition, multiple baseline algorithms will be compared to comprehensively demonstrate the performance of the proposed algorithm.

### 5.1. Experimental Parameter Setting

In our simulation experiment, we established an edge computing environment with two cloud servers, four edge servers, and 20 user devices connected by communication links. Computational tasks were randomly generated by user devices following a Poisson distribution, with an average arrival rate of 30 tasks per second.

For the simulation parameters, the data volume of tasks $\alpha_{i}$ followed a uniform distribution between 0.5 and 1 bit, and the computational load $\beta_{i}$ ranged from 0.3 to 3 Gcycles. The delay sensitivity coefficient of tasks $\gamma_{i}$ was [1, 2, 3], with timeouts uniformly distributed between 100 and 300 ms after task generation. The communication rates were as follows: 4–6 MB/s between the user devices and edge servers, 15–25 MB/s between edge servers, 40–60 MB/s between edge and cloud servers, and 80–120 MB/s between cloud servers. The CPU clock frequencies were 3–4 GHz for the user devices, 60–80 GHz for the edge servers, and 125–175 GHz for the cloud servers.

In addition, the experiment simulated the volatility problem in the edge computing scenario. Cloud servers and edge servers, which are usually centrally managed, have low computational volatility. Due to environmental factors such as temperature fluctuations, an unstable energy supply, and changes in network conditions, the computing power and response time of IoT devices may experience significant fluctuations. Therefore, we set the $\omega_{k}$ of the cloud server, edge server and user devices to 0.1, 0.15, and 0.25 and set $\varphi_{k}$ to 0.02, 0.05, and 0.1, consequently.

The above experimental set-up draws inspiration from related studies [21,22,23], aiming to enhance the efficiency and sustainability of edge computing systems.

### 5.2. Experiment Profile

We first compared the results of our proposed algorithm with several common baseline algorithms within the same testing environment. The baseline algorithms used for comparison are introduced below:

- Local computing: In this scenario, each mobile device processes its own computing tasks locally without offloading. This method enhances responsiveness and reduces latency but may result in longer processing times for resource-intensive tasks, depending on the device’s capabilities.
- Random offloading: This strategy involves randomly assigning computing tasks to either the cloud server or local processing. It is a simple method which does not guarantee optimal resource utilization but serves as a baseline for evaluating other, more complex algorithms.
- Actor-critic: This reinforcement learning strategy combines policy-based and value-based methods. The “actor” suggests actions according to the current policy, while the “critic” evaluates these actions using a value function. This method dynamically adapts decisions, aiming to optimize task offloading through continuous learning, thereby enhancing system efficiency.

**Figure 5:**
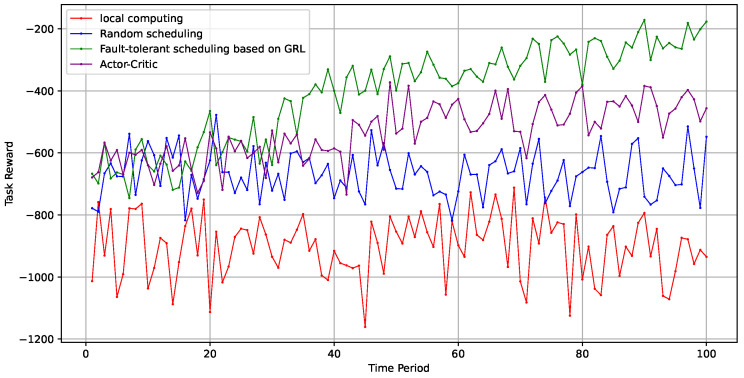
Algorithm comparison.

As shown in Figure 5, the system overhead for stochastic scheduling and local computing remained consistently high. In contrast, the algorithm proposed in this paper demonstrated superior performance, surpassing the traditional actor-critic algorithm.

Since stability is a key metric in the study of fault tolerance within edge computing scenarios, we focused on evaluating the performance of scheduling algorithms across multiple experiments under identical conditions. We then assessed the stability of the fault-tolerant scheduling algorithm through numerous trials, observing that the optimization model maintained a relatively stable optimization process. This indicates that the algorithm possesses strong robustness and adaptability, effectively addressing various challenges in the edge computing environment to ensure service continuity and reliability.

**Figure 6:**
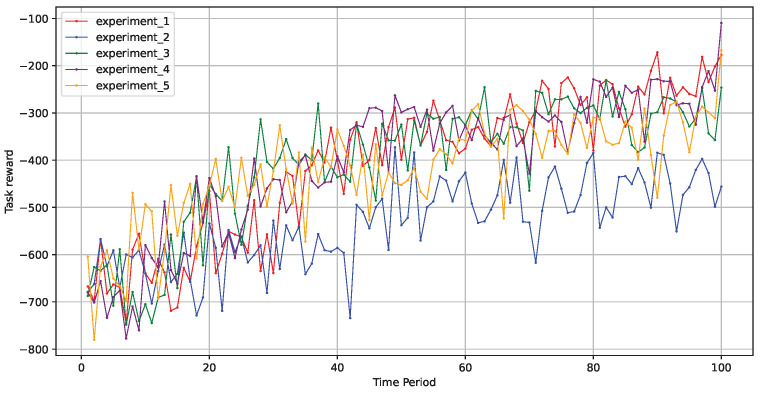
Stability across five experimental rounds.

As illustrated in Figure 6, across five rounds of experiments in different environments, the model effectively improved its learning strategy to make better decisions, and the optimization process remained relatively stable. This demonstrates that the fault-tolerant scheduling model based on a graph neural network, as proposed in this paper, exhibited good stability.

**Figure 7:**
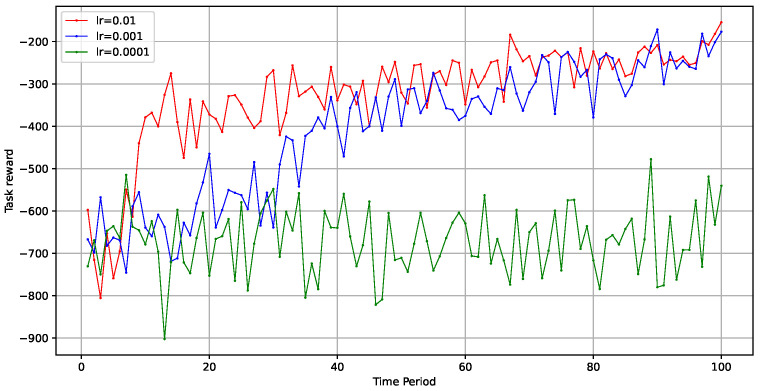
Impact of learning rate on optimization.

Additionally, we explored the impact of the learning rate on the model’s optimization speed and effectiveness. We set the learning rates to 0.01, 0.001, and 0.0001 to evaluate performance when the total number of tasks was 10,000. As shown in Figure 7, the model’s training speed during the early stages accelerated with higher learning rates, while the final optimization outcomes tended to converge similarly. However, when the learning rate was too low, such as 0.0001, the model struggled to find an optimal strategy.

**Figure 8:**
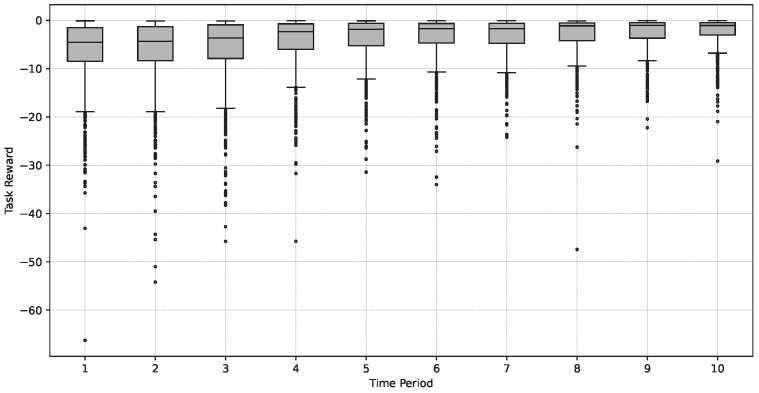
Task cost dispersion and unloading balance.

Furthermore, we examined the degree of task cost dispersion within the model to assess the balance of task offloading among different devices and resources, thereby evaluating the system’s performance and efficiency. Figure 8 displays the quartile distribution of task costs for every 1000 tasks. It is evident from the figure that the task costs gradually decreased, the degree of cost dispersion diminished, and the balance of task offloading strengthened over time.

**Figure 9:**
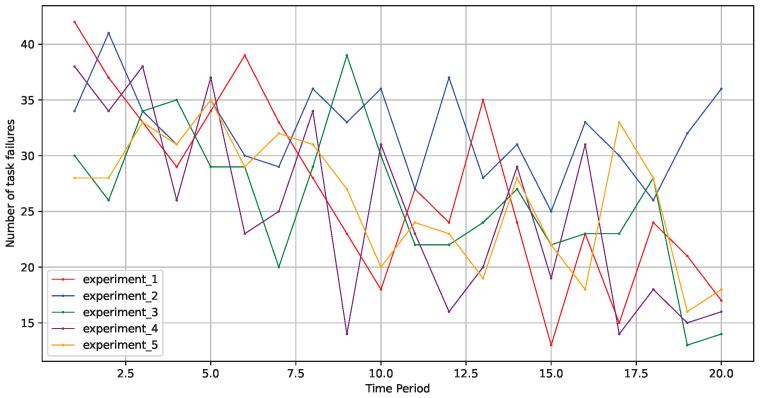
Failure rate trends.

The following figure illustrates the main components contributing to task time delay and the average changes in the failure rate. The failure rate calculation in this study primarily involved the active replication of the model to achieve fault-tolerant scheduling behavior, imposing significant penalties when errors occurred in task computations. Therefore, as depicted in Figure 9, the failure rate exhibited a downward trend as the number of iterations increased.

**Figure 10:**
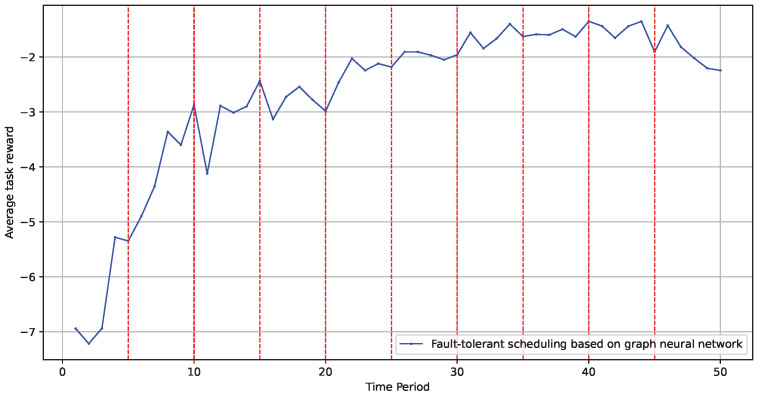
Model performance in dynamic environments.

Finally, we tested the influence of a dynamic environment on the model’s optimization effectiveness. We evaluated whether the model could be continuously updated iteratively within a dynamic topology by periodically altering the graph structure and node information. The experiment was set up by changing the experimental environment graph’s structure every 1000 tasks to test the model’s adaptability to environmental changes. As shown in Figure 10, changes in the edge computing scene and topology structure did not adversely affect the model’s implementation effectiveness. The red dotted line in the figure represents the time when the topological environment is changed.

## 6. Conclusions

In this paper, we examined task scheduling at the finest granularity by treating each task as indivisible to ensure precise management. We proposed a fault-tolerant scheduling algorithm based on graph reinforcement learning to address task flow scheduling in mobile edge computing environments. The algorithm leverages a graph neural network layer built on the message passing neural network (MPNN) framework, incorporating actor and critic architectures within the reinforcement learning model. By processing features effectively, the fault-tolerant strategy is generated as the output of the graph neural network. Combining this with the proximal policy optimization (PPO) update strategy enables stable and efficient model updates.

Simulation experiments comparing our algorithm with several baseline methods demonstrated that our approach consistently achieved stable policy updates and robust performance in highly dynamic and variable scenarios. These results highlight the algorithm’s effectiveness in maintaining reliable task processing and ensuring high user satisfaction in real-world mobile edge computing settings.

Future work will explore more complex task dependencies and heterogeneous resources within mobile edge computing. Enhancements to the graph neural network architecture and the integration of additional reinforcement learning techniques could further improve performance. Additionally, real-world deployment and testing will be essential to validate the algorithm’s practical applicability and resilience in diverse, large-scale environments.

## Figures and Tables

**Table 1:** Comparison of traditional and ML-based approaches in edge computing task scheduling.

Aspect	Traditional Approaches	ML-Based Approaches
Adaptability	Limited; often requires manual reconfiguration	High; can adapt to changing environments
Scalability	Often limited by predefined rules	Generally more scalable to complex scenarios
Computational Overhead	Usually lower	Higher, especially during training phase
Fault Tolerance	Typically rule-based, limited flexibility	Can learn and improve fault handling over time
Resource Utilization	Based on predefined metrics	Can optimize for complex, multi-dimensional objectives
Implementation Complexity	Generally simpler to implement	Requires expertise in ML; more complex set-up
Real-Time Performance	Often faster for simple scenarios	May have latency issues, but potentially better for complex scenarios

## Data Availability

The data presented in this study are available on request from thecorresponding author.

## Abbreviations

The following abbreviations are used in this manuscript:

*n*	Number of available IoT devices in edge computing
$V_{k}$	Model of device *k*, with its parameters
$f_{k}$	Computing capability of device *k*
$\omega_{k}$	Uncertainty in processing tasks on device *k*
$\varphi_{k}$	Task failure rate on device *k*
$R_{m,n}$	Transmission rate from device *m* to device *n*
$\omega_{m,n}$	Uncertainty of transmission rate from *m* to *n*
$T_{i}^{m,n}$	Actual transmission time for task *i* from *m* to *n*
$\alpha_{i}$	Data size of task *i*
$T_{i}^{trans}$	Total transmission time for task *i*
$E_{i}$	Network links traversed by task *i*
$t_{i}$	Generation time of task *i*
$T_{i}^{dec}$	Decision-making time for task *i*
$T_{i}^{wait}$	Waiting time for task *i*
$t_{k,i}$	Start time of task *i* on device *k*
$T_{k,i}$	Computation time for task *i* on device *k*
$\beta_{i}$	Computational demand of task *i*
$T_{k,i}^{actual}$	Actual computation time for task *i* on device *k*
$t_{k,i}^{finish}$	Finishing time of task *i* on device *k*
$t_{i}^{deadline}$	Deadline of task *i*
$T_{k,i}^{timeout}$	Timeout period for task *i*
$\kappa_{k,i}$	Timeout limit for failed task *i* on device *k*
$\gamma_{i}$	Timeout weight for task *i*
N	Set of all computing tasks
